# Supplementary material for: Reference genes for gene expression analysis in the fungal pathogen Neonectria ditissima and their use demonstrating expression up-regulation of candidate virulence genes
Source: PLoS One. 2020 Nov 13;15(11):e0238157. doi: 10.1371/journal.pone.0238157 (PMC7665675; doi:10.1371/journal.pone.0238157)

Fig 1 original gel images

actin. M = 1kb Plus DNA Ladder (Invitrogen^{TM}, Thermo Fisher Scientific, MA, USA). Amplification using gDNA template from (1) *Botryosphaeria* sp., (2) *Cladosporium* sp., (3) *Colletotrichum acutatum*, (4) *Colletotrichum gloeosporioides*, (5) *Neofabraea alba*, (6) *Neofabraea malicortis*, (7) *Neofabraea perennans*, (8) *Venturia inaequalis* 1639, (9) *Venturia inaequalis* MNH120, (10) *Venturia inaequalis* EUB04, (11) *N. ditissima* RS324p as a positive control and (-) non-template negative control.

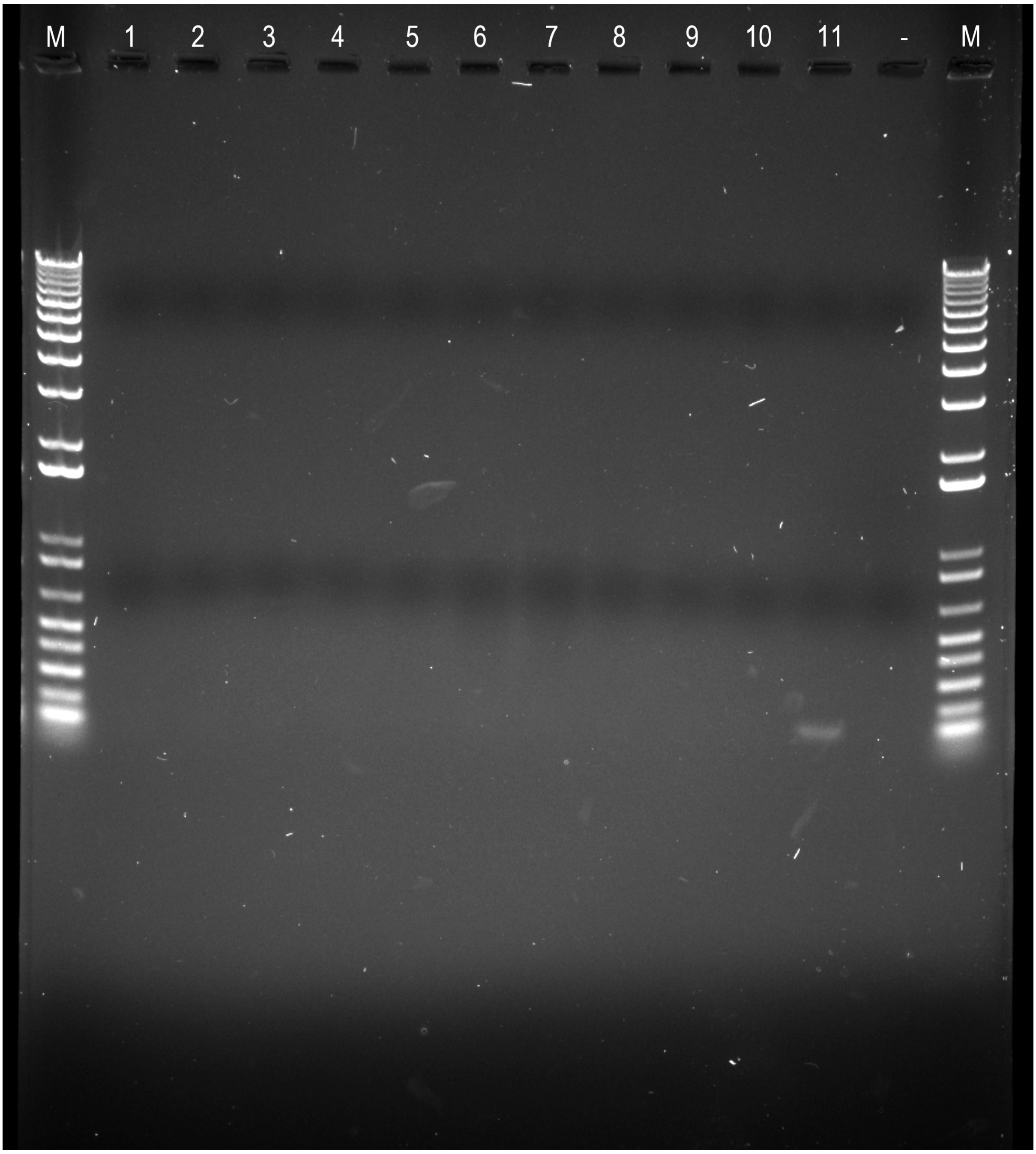

gapdh. M = 1kb Plus DNA Ladder (Invitrogen™, Thermo Fisher Scientific, MA, USA). Amplification using gDNA template from (1) *Botryosphaeria* sp., (2) *Cladosporium* sp., (3) *Colletotrichum acutatum*, (4) *Colletotrichum gloeosporioides*, (5) *Neofabraea alba*, (6) *Neofabraea malicortis*, (7) *Neofabraea perennans*, (8) *Venturia inaequalis* 1639, (9) *Venturia inaequalis* MNH120, (10) *Venturia inaequalis* EUB04, (11) *N. ditissima* RS324p as a positive control and (-) non-template negative control.

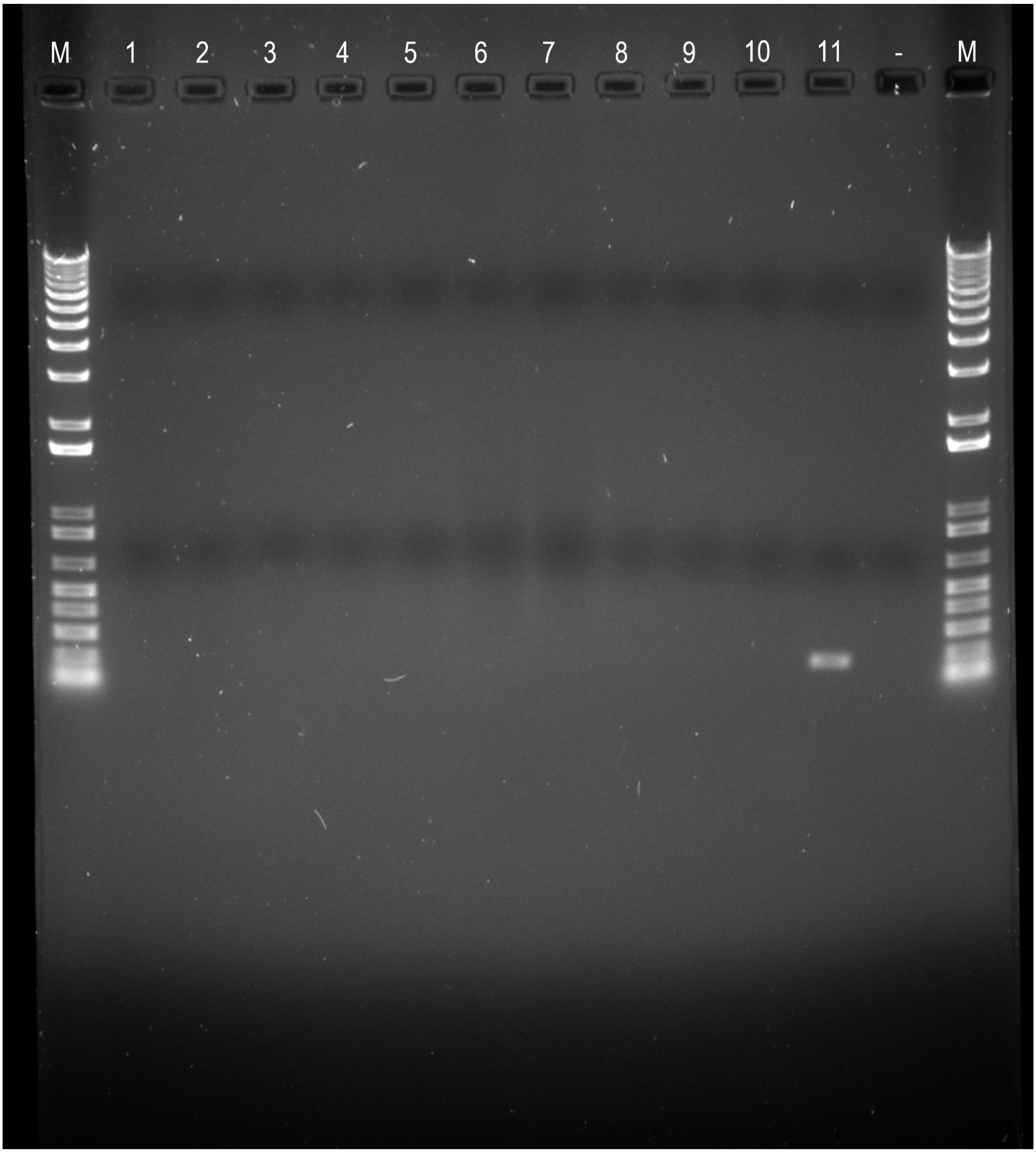

S8. M = 1kb Plus DNA Ladder (Invitrogen™, Thermo Fisher Scientific, MA, USA). Amplification using gDNA template from (1) *Botryosphaeria* sp., (2) *Cladosporium* sp., (3) *Colletotrichum acutatum*, (4) *Colletotrichum gloeosporioides*, (5) *Neofabraea alba*, (6) *Neofabraea malicortis*, (7) *Neofabraea perennans*, (8) *Venturia inaequalis* 1639, (9) *Venturia inaequalis* MNH120, (10) *Venturia inaequalis* EUB04, (11) *N. ditissima* RS324p as a positive control and (-) non-template negative control.

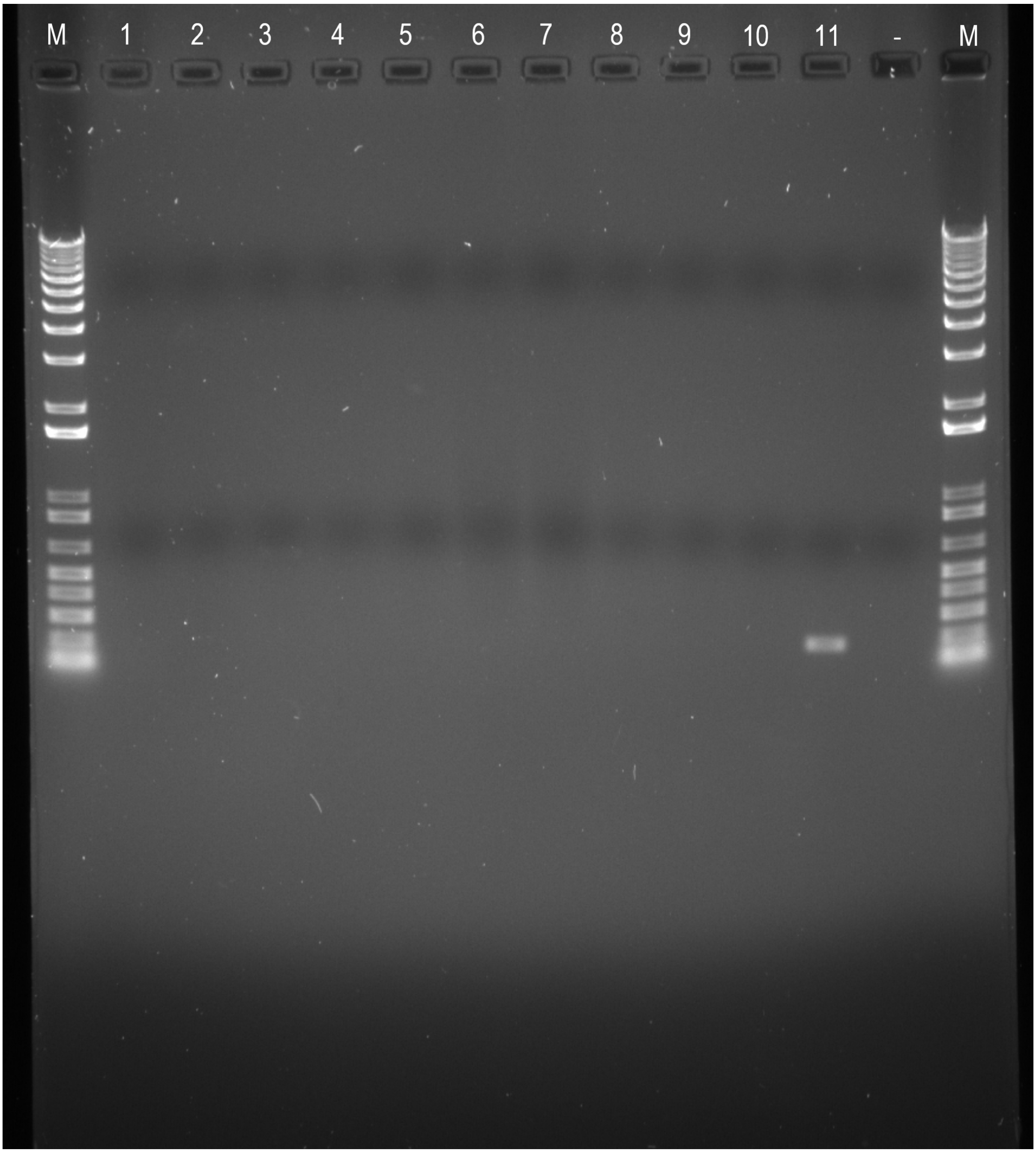

18sAMT. M = 1kb Plus DNA Ladder (Invitrogen^{TM}, Thermo Fisher Scientific, MA, USA). Amplification using gDNA template from (1) *Botryosphaeria* sp., (2) *Cladosporium* sp., (3) *Colletotrichum acutatum*, (4) *Colletotrichum gloeosporioides*, (5) *Neofabraea alba*, (6) *Neofabraea malicortis*, (7) *Neofabraea perennans*, (8) *Venturia inaequalis* 1639, (9) *Venturia inaequalis* MNH120, (10) *Venturia inaequalis* EUB04, (11) *N. ditissima* RS324p as a positive control and (-) non-template negative control.

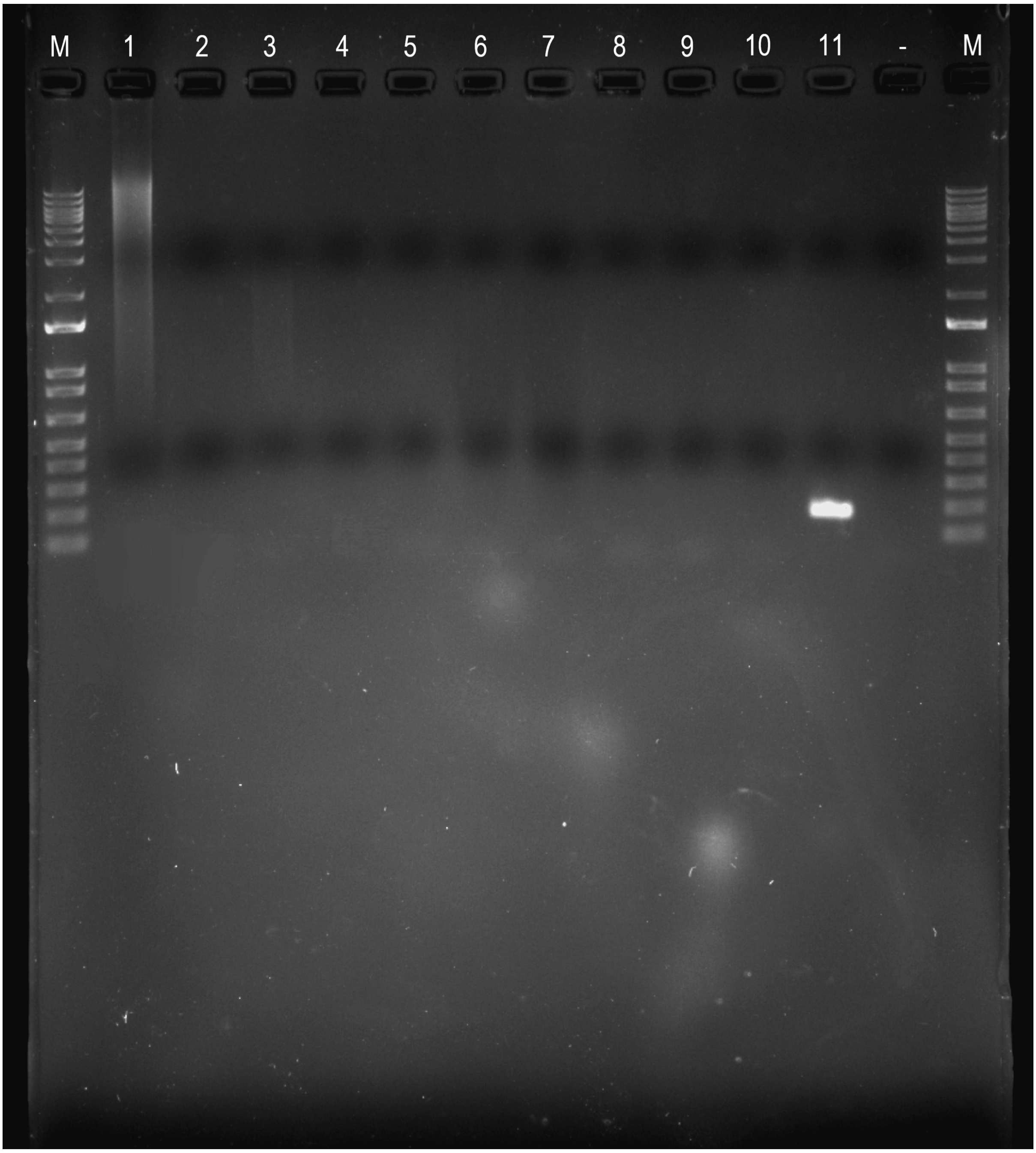

Btub. M = 1kb Plus DNA Ladder (Invitrogen™, Thermo Fisher Scientific, MA, USA). Amplification using gDNA template from (1) *Botryosphaeria* sp., (2) *Cladosporium* sp., (3) *Colletotrichum acutatum*, (4) *Colletotrichum gloeosporioides*, (5) *Neofabraea alba*, (6) *Neofabraea malicortis*, (7) *Neofabraea perennans*, (8) *Venturia inaequalis* 1639, (9) *Venturia inaequalis* MNH120, (10) *Venturia inaequalis* EUB04, (11) *N. ditissima* RS324p as a positive control and (-) non-template negative control.

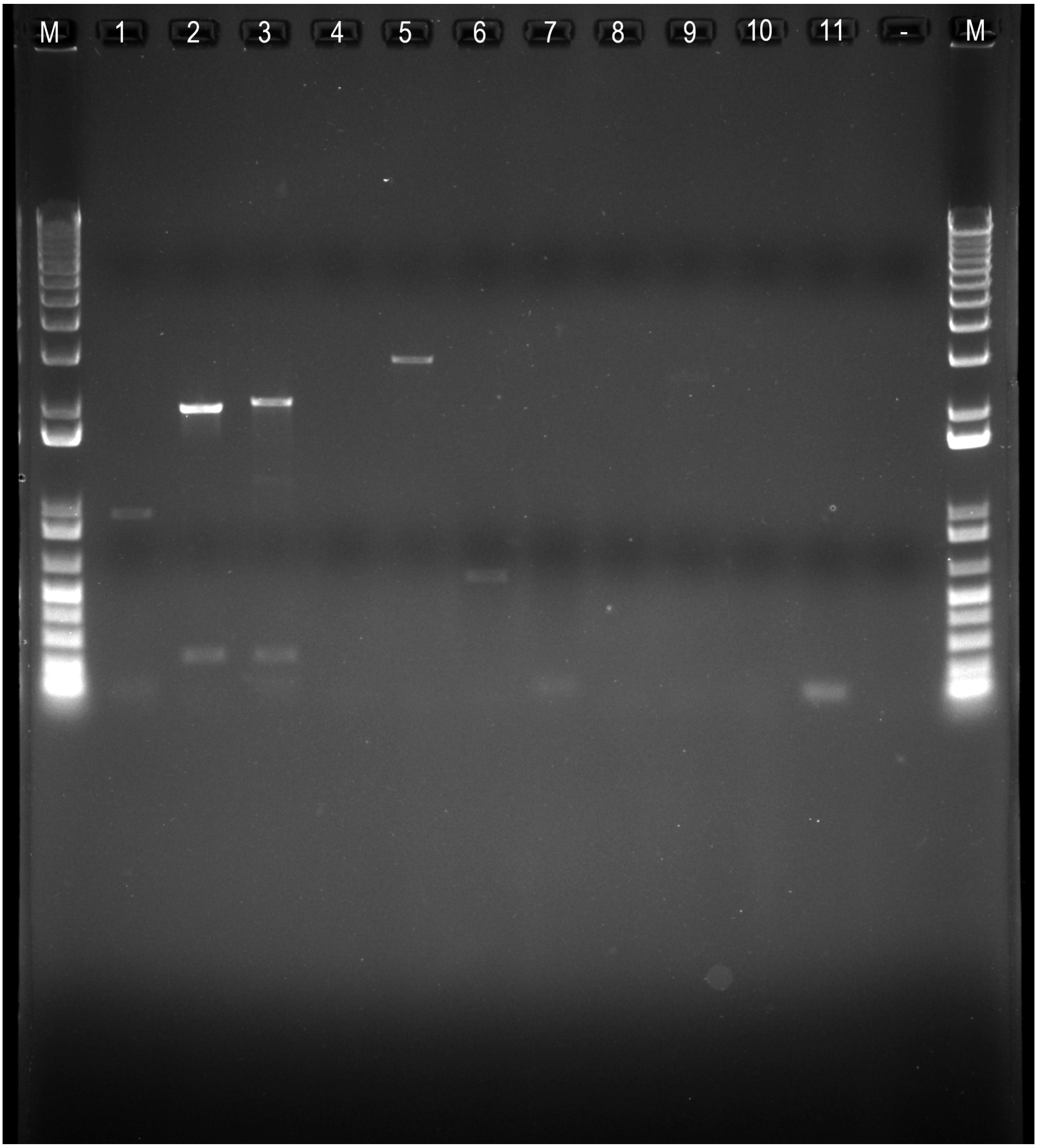

EfTu. M = 1kb Plus DNA Ladder (Invitrogen^{TM}, Thermo Fisher Scientific, MA, USA). Amplification using gDNA template from (1) *Botryosphaeria* sp., (2) *Cladosporium* sp., (3) *Colletotrichum acutatum*, (4) *Colletotrichum gloeosporioides*, (5) *Neofabraea alba*, (6) *Neofabraea malicortis*, (7) *Neofabraea perennans*, (8) *Venturia inaequalis* 1639, (9) *Venturia inaequalis* MNH120, (10) *Venturia inaequalis* EUB04, (11) *N. ditissima* RS324p as a positive control and (-) non-template negative control.

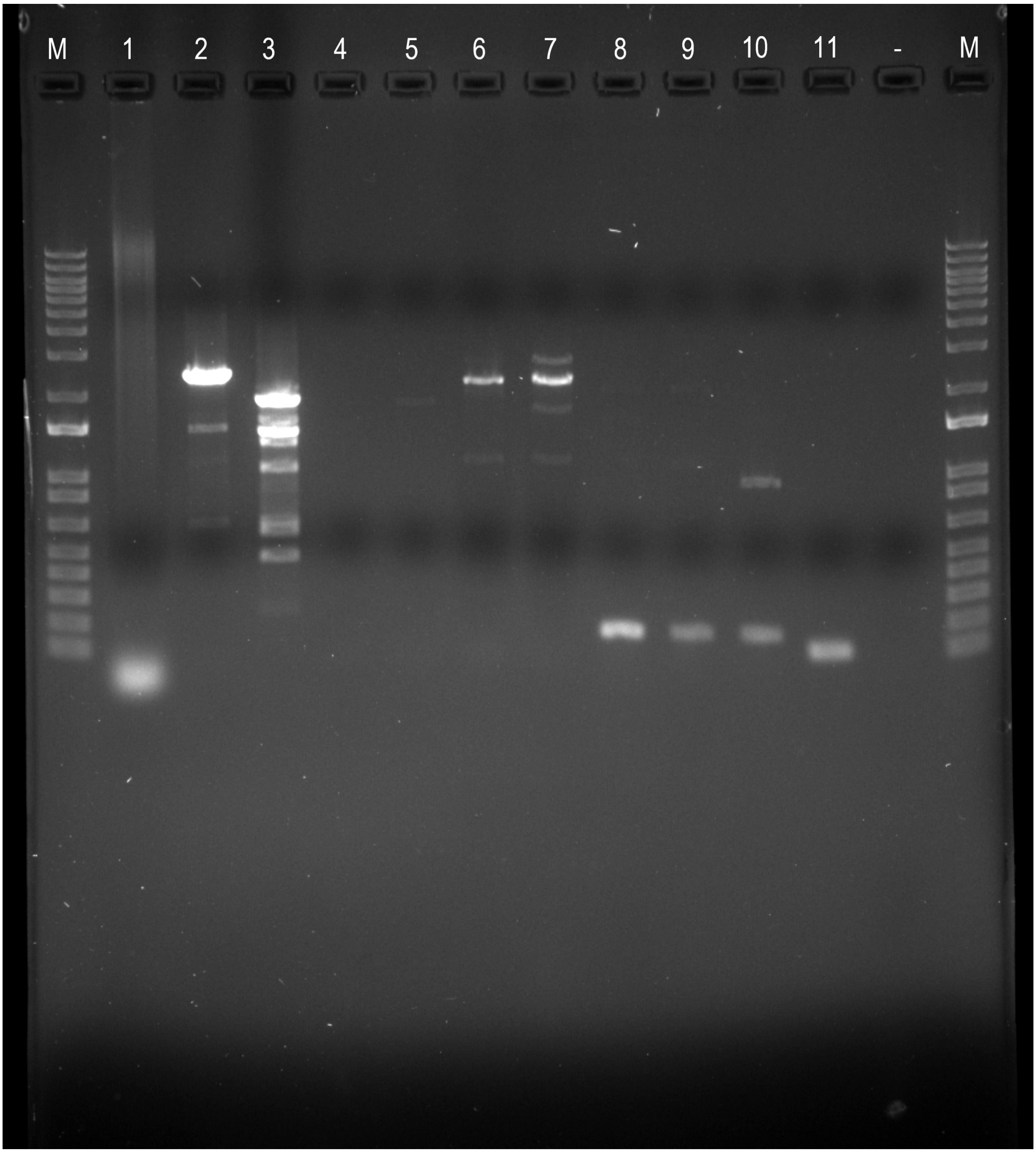

E2. M = 1kb Plus DNA Ladder (Invitrogen™, Thermo Fisher Scientific, MA, USA). Amplification using gDNA template from (1) *Botryosphaeria* sp., (2) *Cladosporium* sp., (3) *Colletotrichum acutatum*, (4) *Colletotrichum gloeosporioides*, (5) *Neofabraea alba*, (6) *Neofabraea malicortis*, (7) *Neofabraea perennans*, (8) *Venturia inaequalis* 1639, (9) *Venturia inaequalis* MNH120, (10) *Venturia inaequalis* EUB04, (11) *N. ditissima* RS324p as a positive control and (-) non-template negative control.

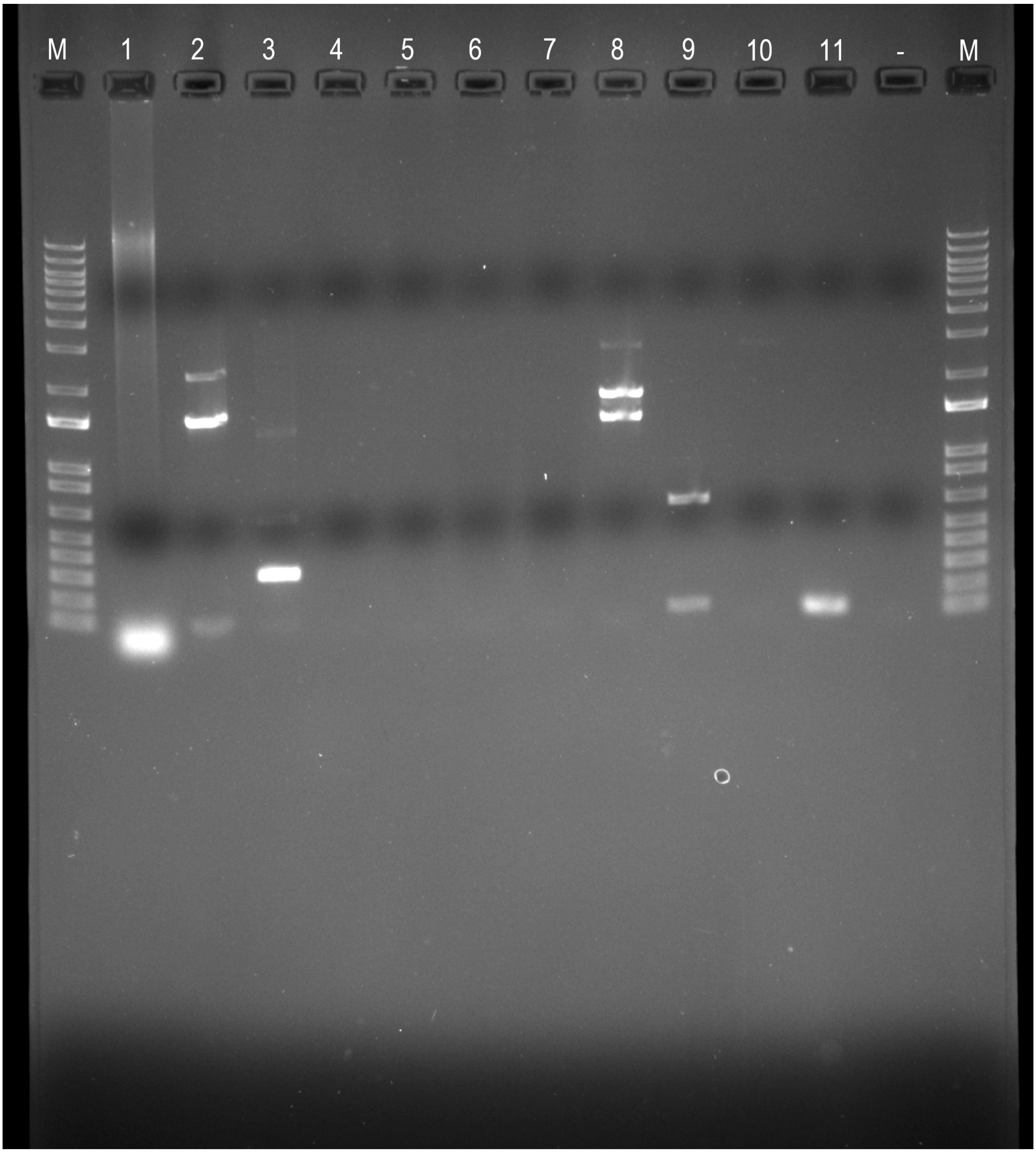

S27a. M = 1kb Plus DNA Ladder (Invitrogen™, Thermo Fisher Scientific, MA, USA). Amplification using gDNA template from (1) *Botryosphaeria* sp., (2) *Cladosporium* sp., (3) *Colletotrichum acutatum*, (4) *Colletotrichum gloeosporioides*, (5) *Neofabraea alba*, (6) *Neofabraea malicortis*, (7) *Neofabraea perennans*, (8) *Venturia inaequalis* 1639, (9) *Venturia inaequalis* MNH120, (10) *Venturia inaequalis* EUB04, (11) *N. ditissima* RS324p as a positive control and (-) non-template negative control.

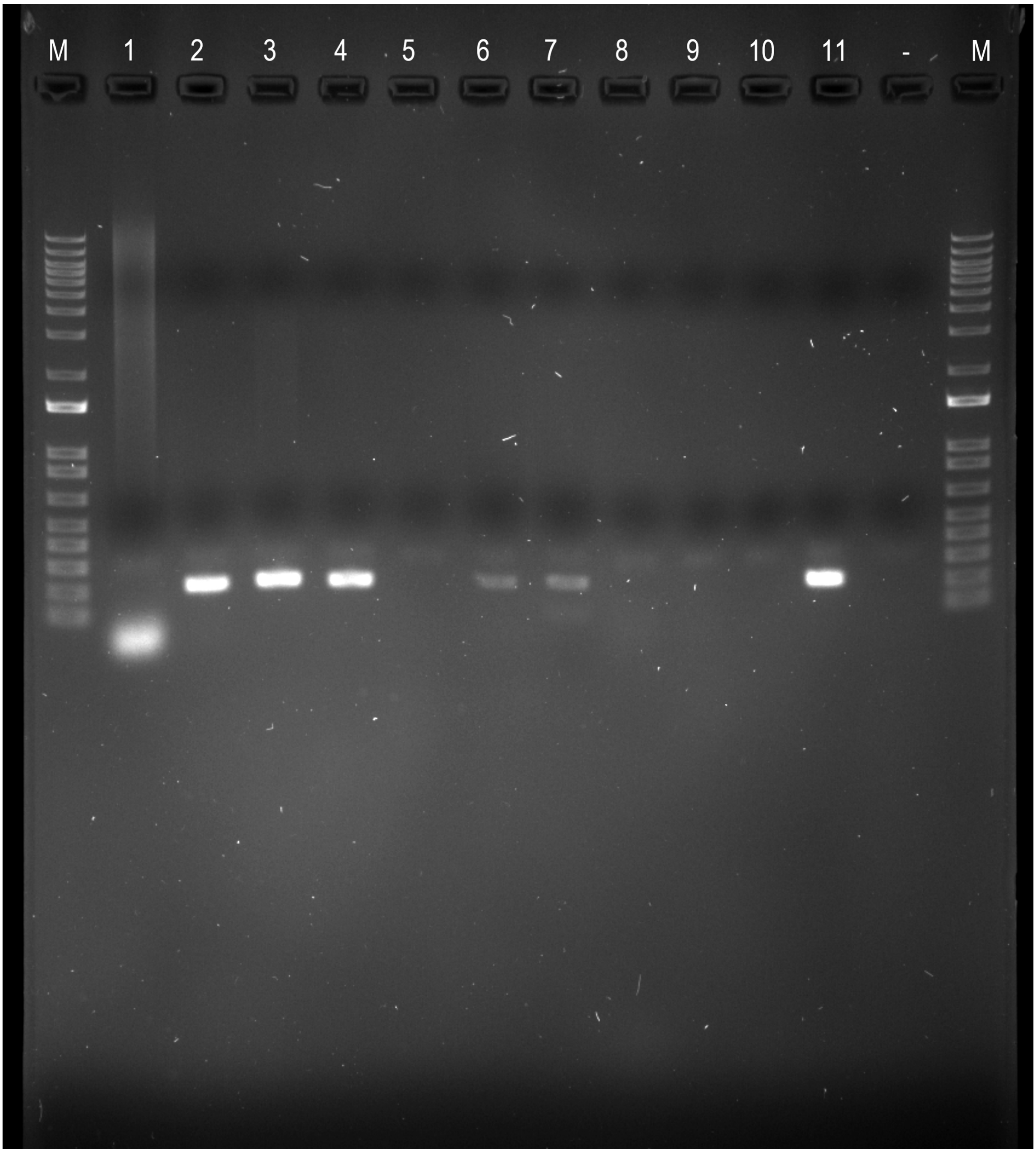

Supplement: S1 Fig — (PDF) [file pone.0238157.s001.pdf]
