# Supplementary figures and images for: Reference genes for gene expression analysis in the fungal pathogen Neonectria ditissima and their use demonstrating expression up-regulation of candidate virulence genes
Source: PLoS One. 2020 Nov 13;15(11):e0238157. doi: 10.1371/journal.pone.0238157 (PMC7665675; doi:10.1371/journal.pone.0238157)

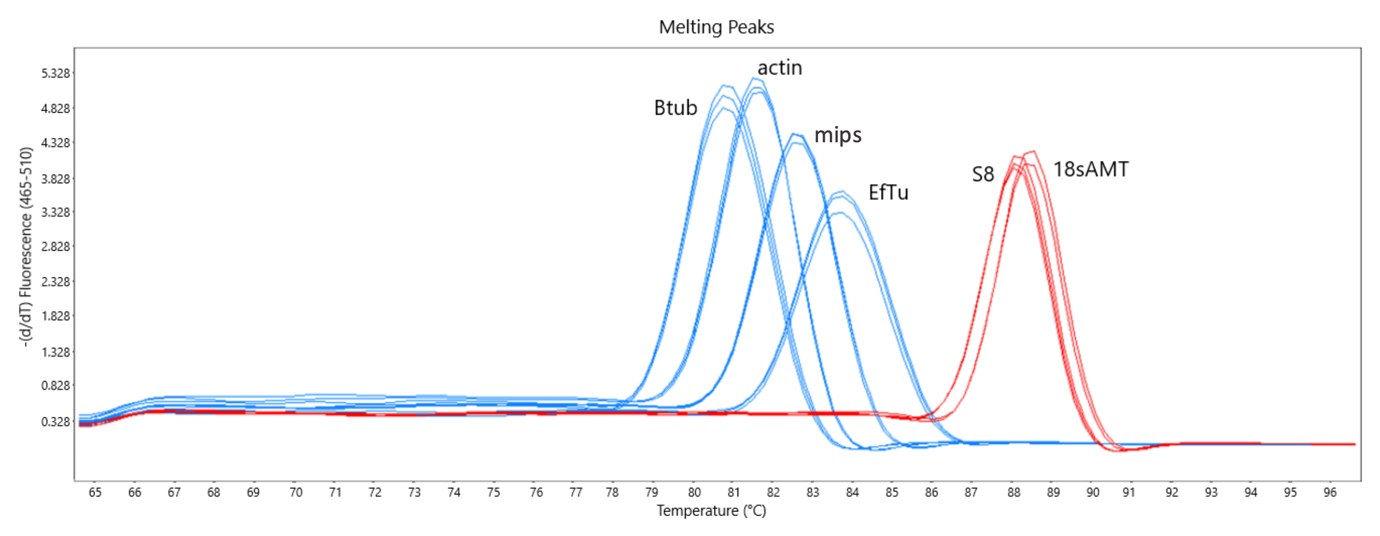

Supplement: S2 Fig — Single peaks were observed at the melting temperature (°C) of the respective amplicons. actin—81.6, Btub—80.9, mips—82.7, EfTu—83.8, S8–88.0, 18sAMT—88.5. (TIF) [file pone.0238157.s002.tif]

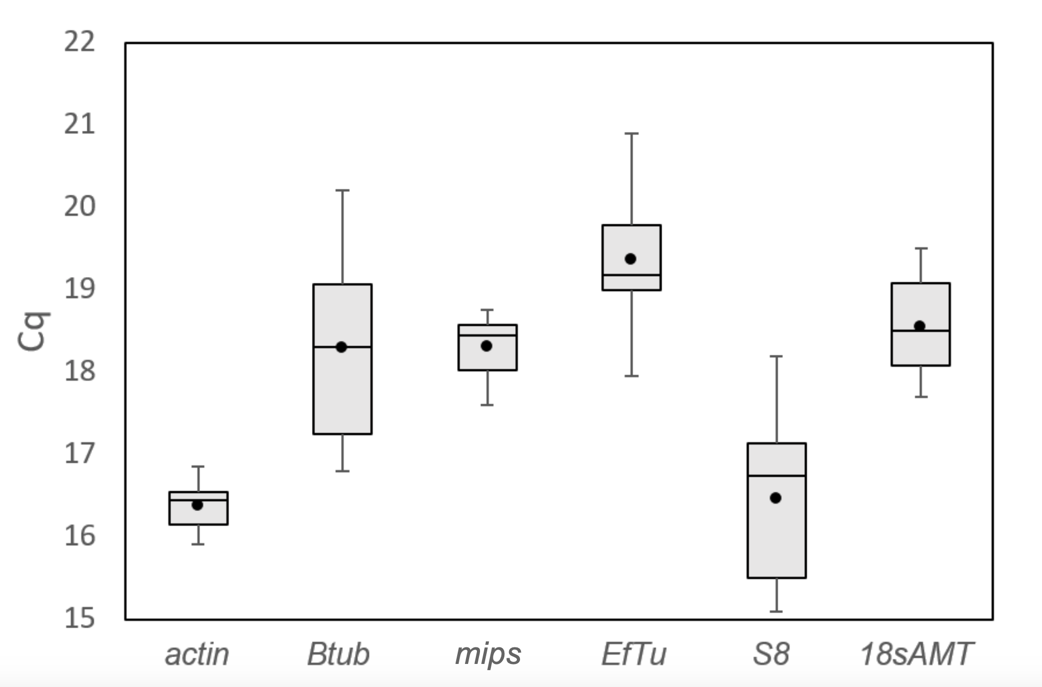

Supplement: S3 Fig — Data derived from three technical replicates from three biological replicates. (TIF) [file pone.0238157.s003.tif]

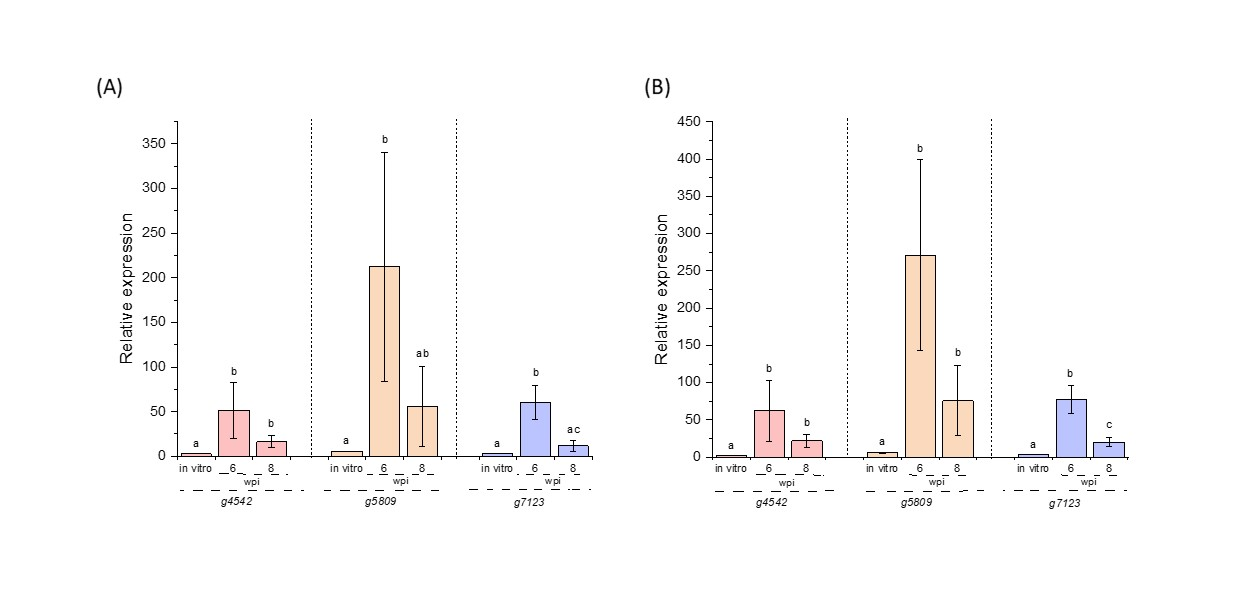

Supplement: S4 Fig — The relative expression of three Neonectria ditissima candidate virulence genes using (A) the least stable reference genes S8 and Btub versus (B) the most stably expressed reference genes actin and mips. Data derived from three technical replicates from three biological replicates. (TIF) [file pone.0238157.s004.tif]
