# Supplementary material for: Reference genes for gene expression analysis in the fungal pathogen Neonectria ditissima and their use demonstrating expression up-regulation of candidate virulence genes
Source: PLoS One. 2020 Nov 13;15(11):e0238157. doi: 10.1371/journal.pone.0238157 (PMC7665675; doi:10.1371/journal.pone.0238157)
Supplement: S1 Table — (DOCX) [file pone.0238157.s005.docx]

**S1 Table. Functional similarities of candidate reference and virulence genes revealed by BLASTn and BLASTp searches against the *Neonectria ditissima* R09/05 genome database in the** [**MycoCosm**](https://mycocosm.jgi.doe.gov/pages/blast-query.jsf?db=Neodi1) **website**.

|  | | ID | e value | Gene function | Link |
| --- | --- | --- | --- | --- | --- |
| *actin* | gene | Neodi1\|7315 | 0.00E+00 | actin and related proteins | [^[1]^](https://mycocosm.jgi.doe.gov/cgi-bin/dispGeneModel?db=Neodi1&tid=7315) |
|  | protein | Neodi1\|7315 | 0.00E+00 | actin and related proteins | [^[2]^](https://mycocosm.jgi.doe.gov/pages/blast-results.jsf;ofMWZi?groupId=1486127&db=Neodi1) |
| *mips* | gene | Neodi1\|4374 | 0.00E+00 | myo-inositol-1-phosphate-synthase | [^[3]^](https://mycocosm.jgi.doe.gov/cgi-bin/dispGeneModel?db=Neodi1&tid=4374) |
|  | protein | Neodi1\|4374 | 0.00E+00 | myo-inositol-1-phosphate synthase | [^[4]^](https://mycocosm.jgi.doe.gov/cgi-bin/dispGeneModel?db=Neodi1&id=4374) |
| *S8* | gene | Neodi1\|6284 | 0.00E+00 | Ribosomal protein S8e | [^[5]^](https://mycocosm.jgi.doe.gov/cgi-bin/dispGeneModel?db=Neodi1&tid=6284) |
|  | protein | Neodi1\|6284 | 4.85E-129 | Ribosomal protein S8e | [^[6]^](https://mycocosm.jgi.doe.gov/cgi-bin/dispGeneModel?db=Neodi1&id=6284) |
| *18sAMT* | gene | Neodi1\|2517 | 0.00E+00 | Ribosomal RNA adenine methylase transferase | [^[7]^](https://mycocosm.jgi.doe.gov/cgi-bin/dispGeneModel?db=Neodi1&tid=2517) |
|  | protein | Neodi1\|2517 | 0.00E+00 | Ribosomal RNA adenine methylase transferase | [^[8]^](https://mycocosm.jgi.doe.gov/cgi-bin/dispGeneModel?db=Neodi1&id=2517) |
| *btub* | gene | Neodi1\|9151 | 0.00E+00 | Beta tubulin | [^[9]^](https://mycocosm.jgi.doe.gov/cgi-bin/dispGeneModel?db=Neodi1&tid=9151) |
|  | protein | Neodi1\|9151 | 0.00E+00 | Beta tubulin | [^[10]^](https://mycocosm.jgi.doe.gov/cgi-bin/dispGeneModel?db=Neodi1&id=9151) |
| *EfTu* | gene | Neodi1\|4147 | 0.00E+00 | Translation elongation factor Tu | [^[11]^](https://mycocosm.jgi.doe.gov/cgi-bin/dispGeneModel?db=Neodi1&tid=4147) |
|  | protein | Neodi1\|4147 | 0.00E+00 | Translation elongation factor Tu | [^[12]^](https://mycocosm.jgi.doe.gov/cgi-bin/dispGeneModel?db=Neodi1&id=4147) |
| *E2* | gene | Neodi1\|590 | 0.00E+00 | Ubiquitin-conjugating enzyme | [^[13]^](https://mycocosm.jgi.doe.gov/cgi-bin/dispGeneModel?db=Neodi1&tid=590) |
|  | protein | Neodi1\|590 | 0.00E+00 | Ubiquitin-conjugating enzyme | [^[14]^](https://mycocosm.jgi.doe.gov/cgi-bin/dispGeneModel?db=Neodi1&id=590) |
| *S27a* | gene | Neodi1\|9075 | 0.00E+00 | Ribosomal protein S27a | [^[15]^](https://mycocosm.jgi.doe.gov/cgi-bin/dispGeneModel?db=Neodi1&tid=9075) |
|  | protein | Neodi1\|9075 | 1.78E-81 | Ribosomal protein S27a | [^[16]^](https://mycocosm.jgi.doe.gov/pages/blast-results.jsf;g2MXgR?groupId=1486165&db=Neodi1) |
| *g4542* | gene | Neodi1\|8795 | 0.00E+00 | hypothetical protein | [^[17]^](https://mycocosm.jgi.doe.gov/cgi-bin/dispGeneModel?db=Neodi1&tid=8795) |
|  | protein | Neodi1\|8795 | 4.04E-112 | hypothetical protein | [^[18]^](https://mycocosm.jgi.doe.gov/cgi-bin/dispGeneModel?db=Neodi1&id=8795) |
| *g5809* | gene | Neodi1\|11801 | 0.00E+00 | hypothetical protein | [^[19]^](https://mycocosm.jgi.doe.gov/cgi-bin/dispGeneModel?db=Neodi1&tid=11801) |
|  | protein | Neodi1\|11801 | 3.42E-123 | hypothetical protein | [^[20]^](https://mycocosm.jgi.doe.gov/cgi-bin/dispGeneModel?db=Neodi1&id=11801) |
| *g7123* | gene | Neodi1\|9845 | 0.00E+00 | hypothetical protein | [^[21]^](https://mycocosm.jgi.doe.gov/cgi-bin/dispGeneModel?db=Neodi1&tid=9845) |
|  | protein | Neodi1\|9845 | 4.38E-99 | hypothetical protein | [^[22]^](https://mycocosm.jgi.doe.gov/cgi-bin/dispGeneModel?db=Neodi1&id=9845) |

^1^ Threshold for similarity: e-10
