# Supplementary material for: Reference genes for gene expression analysis in the fungal pathogen Neonectria ditissima and their use demonstrating expression up-regulation of candidate virulence genes
Source: PLoS One. 2020 Nov 13;15(11):e0238157. doi: 10.1371/journal.pone.0238157 (PMC7665675; doi:10.1371/journal.pone.0238157)
Supplement: S2 Table — (DOCX) [file pone.0238157.s006.docx]

**S2 Table**. **Functional similarities of candidate reference and virulence genes revealed by BLASTn and BLASTp searches against the databases of reference RNA sequences (refseq_rna) and reference protein sequences (refseq_protein) respectively, in NCBI****.**

|  |  | Function of most similar gene/protein | Organism | e value ^1^ | Accession number |
| --- | --- | --- | --- | --- | --- |
| *actin* | gene | actin | *Fusarium graminearum* | 0.00E+00 | [XM_011328784.1](https://www.ncbi.nlm.nih.gov/nucleotide/XM_011328784.1?report=genbank&log$=nucltop&blast_rank=4&RID=8U6JS3DB014) |
|  | protein | actin | *Verticillium alfalfae* | 0.00E+00 | [XP_003008477.1](https://www.ncbi.nlm.nih.gov/protein/XP_003008477.1?report=genbank&log$=prottop&blast_rank=1&RID=8U6NU2RU01R) |
| *mips* | gene | predicted protein | *Nectria haematococca* | 0.00E+00 | [XM_003051114.1](https://www.ncbi.nlm.nih.gov/nucleotide/XM_003051114.1?report=genbank&log$=nucltop&blast_rank=1&RID=8U8TNN0P014) |
|  | protein | predicted protein | *Nectria haematococca* | 0.00E+00 | [XP_003051160.1](https://www.ncbi.nlm.nih.gov/protein/XP_003051160.1?report=genbank&log$=protalign&blast_rank=1&RID=8U8U7F98014) |
| *S8* | gene | 40S ribosomal protein S8 | *Nectria haematococca* | 0.00E+00 | [XM_003054393.1](https://www.ncbi.nlm.nih.gov/nucleotide/XM_003054393.1?report=genbank&log$=nucltop&blast_rank=1&RID=8U9X2NSG014) |
|  | protein | 40S ribosomal protein S9 | *Nectria haematococca* | 1E-139 | [XP_003054439.1](https://www.ncbi.nlm.nih.gov/protein/XP_003054439.1?report=genbank&log$=prottop&blast_rank=1&RID=8U9WVKBX016) |
| *18sAMT* | gene | dimethyl adenosine transferase | *Purpureocillium lilacinum* | 0.00E+00 | [XM_018325564.1](https://www.ncbi.nlm.nih.gov/nucleotide/XM_018325564.1?report=genbank&log$=nucltop&blast_rank=1&RID=8UAH0SYA014) |
|  | protein | dimethyl adenosine transferase | *Purpureocillium lilacinum* | 0.00E+00 | [XP_018175316.1](https://www.ncbi.nlm.nih.gov/protein/XP_018175316.1?report=genbank&log$=prottop&blast_rank=1&RID=8UAHES4C014) |
| *btub* | gene | beta-tubulin (TUB2) | *Fusarium euwallaceae* | 0.00E+00 | [KU171782.1](https://www.ncbi.nlm.nih.gov/nucleotide/KU171782.1?report=genbank&log$=nucltop&blast_rank=2&RID=8UAY8X7V016) |
|  | protein | tubulin beta chain | *Fusarium verticillioides* | 0.00E+00 | [XP_018748359.](https://www.ncbi.nlm.nih.gov/protein/XP_018748359.1?report=genbank&log$=prottop&blast_rank=1&RID=8UAYUB1P016) |
| *EfTu* | gene | elongation factor 1-alpha | *Fusarium oxysporum* | 0.00E+00 | [XM_031178713.1](https://www.ncbi.nlm.nih.gov/nucleotide/XM_031178713.1?report=genbank&log$=nucltop&blast_rank=4&RID=8UBH868X014) |
|  | protein | uncharacterised protein | *Fusarium coffeatum* | 0.00E+00 | [XP_031018674.1](https://www.ncbi.nlm.nih.gov/protein/XP_031018674.1?report=genbank&log$=prottop&blast_rank=2&RID=8UBMS8GU016) |
| *E2* | gene | predicted protein | *Nectria haematococca* | 0.00E+00 | [XM_003053378.1](https://www.ncbi.nlm.nih.gov/nucleotide/XM_003053378.1?report=genbank&log$=nucltop&blast_rank=1&RID=8UBZ9B4H016) |
|  | protein | ubiquitin-conjugating enzyme | *Fusarium proliferatum* | 0.00E+00 | [XP_031075730.1](https://www.ncbi.nlm.nih.gov/protein/XP_031075730.1?report=genbank&log$=prottop&blast_rank=3&RID=8UBXE02U014) |
| *S27a* | gene | ubiquitin-40S ribosomal protein S27a | *Fusarium oxysporum* | 0.00E+00 | [XM_031187733.1](https://www.ncbi.nlm.nih.gov/nucleotide/XM_031187733.1?report=genbank&log$=nucltop&blast_rank=3&RID=8UC74U1Y016) |
|  | protein | ubiquitin-40S ribosomal protein S31 | *Nectria haematococca* | 0.00E+00 | [XP_003047931.1](https://www.ncbi.nlm.nih.gov/protein/XP_003047931.1?report=genbank&log$=prottop&blast_rank=1&RID=8UC9CKY0014) |
| *g4542* | gene | None | n/a | n/a | [^[1]^](https://blast.ncbi.nlm.nih.gov/Blast.cgi) |
|  | protein | None | n/a | n/a | [^[2]^](https://blast.ncbi.nlm.nih.gov/Blast.cgi) |
| *g5809* | gene | uncharacterised protein | *Phialemoniopsis curvata* | 1E-88 | [XM_031138108.1](https://www.ncbi.nlm.nih.gov/nucleotide/XM_031138108.1?report=genbank&log$=nucltop&blast_rank=1&RID=8UHD6EVU016) |
|  | protein | hypothetical protein | *Scedosporium apiospermum* | 2E-96 | [XP_016644810.1](https://www.ncbi.nlm.nih.gov/protein/XP_016644810.1?report=genbank&log$=prottop&blast_rank=1&RID=8UJ0TDC7014) |
| *g7123* | gene | hypothetical protein | *Fusarium oxysporum* | 4E-112 | [XM_018380180.1](https://www.ncbi.nlm.nih.gov/nucleotide/XM_018380180.1?report=genbank&log$=nucltop&blast_rank=1&RID=8UJMV299016) |
|  | protein | hypothetical protein | *Fusarium oxysporum* | 5E-93 | [XP_018236394.1](https://www.ncbi.nlm.nih.gov/protein/XP_018236394.1?report=genbank&log$=prottop&blast_rank=1&RID=8UJE7MH3016) |

^1^ Threshold for similarity: e-10
