# Supplementary material for: Reference genes for gene expression analysis in the fungal pathogen Neonectria ditissima and their use demonstrating expression up-regulation of candidate virulence genes
Source: PLoS One. 2020 Nov 13;15(11):e0238157. doi: 10.1371/journal.pone.0238157 (PMC7665675; doi:10.1371/journal.pone.0238157)
Supplement: S3 Table — (DOCX) [file pone.0238157.s007.docx]

**S3 Table**. **Potential protein domains found in the candidate reference and virulence genes when searched with InterProScan5.**

|  | Protein domains | Link |
| --- | --- | --- |
| *actin* | Actin conserved site - ATPase, nucleotide binding domain | [^[1]^](http://www.ebi.ac.uk/Tools/services/rest/iprscan5/result/iprscan5-I20200408-040213-0070-54941658-p2m/svg) |
| *mips* | Myo-inositol-1-phosphate synthase - GAPDH-like (C-terminal) | [^[2]^](http://www.ebi.ac.uk/Tools/services/rest/iprscan5/result/iprscan5-I20200408-035331-0806-53509900-p1m/svg) |
| *S8* | Ribosomal protein S8e | [^[3]^](http://www.ebi.ac.uk/Tools/services/rest/iprscan5/result/iprscan5-I20200408-034913-0399-30349746-p1m/svg) |
| *18sAMT* | Ribosomal RNA adenine methyltransferase | [^[4]^](http://www.ebi.ac.uk/Tools/services/rest/iprscan5/result/iprscan5-I20200408-034328-0437-33122164-p1m/svg) |
| *btub* | Beta-tubulin domain - ribosomal protein L6, alpha-beta domain (C-terminal) | [^[5]^](http://www.ebi.ac.uk/Tools/services/rest/iprscan5/result/iprscan5-I20200408-033756-0958-49582545-p1m/svg) |
| *EfTu* | Elongation factor Tu GTP-binding domain (N-terminal) | [^[6]^](http://www.ebi.ac.uk/Tools/services/rest/iprscan5/result/iprscan5-I20200408-032947-0664-85203349-p2m/svg) |
| *E2* | Ubiquitin-conjugating enzyme E2 (domains at both N and C-terminal) | [^[7]^](http://www.ebi.ac.uk/Tools/services/rest/iprscan5/result/iprscan5-I20200408-032348-0860-47447418-p2m/svg) |
| *S27a* | Ribosomal protein S27a (C-terminal) - Ubiquitin-like domain (N-terminal) | [^[8]^](http://www.ebi.ac.uk/Tools/services/rest/iprscan5/result/iprscan5-I20200408-031047-0472-86506208-p2m/svg) |
| *g4542* | non-cytoplasmic domain (C-terminal) - Signal peptide C/H/N region | [^[9]^](http://www.ebi.ac.uk/Tools/services/rest/iprscan5/result/iprscan5-I20200408-043220-0946-95829864-p1m/svg) |
| *g5809* | non-cytoplasmic domain (C-terminal) - Signal peptide C/H/N region | [^[10]^](http://www.ebi.ac.uk/Tools/services/rest/iprscan5/result/iprscan5-I20200408-044255-0925-22014208-p2m/svg) |
| *g7123* | non-cytoplasmic domain (C-terminal) - Signal peptide C/H/N region | [^[11]^](http://www.ebi.ac.uk/Tools/services/rest/iprscan5/result/iprscan5-I20200408-045236-0190-23313813-p1m/svg) |
