# Supplementary material for: Reference genes for gene expression analysis in the fungal pathogen Neonectria ditissima and their use demonstrating expression up-regulation of candidate virulence genes
Source: PLoS One. 2020 Nov 13;15(11):e0238157. doi: 10.1371/journal.pone.0238157 (PMC7665675; doi:10.1371/journal.pone.0238157)
Supplement: S4 Table — (DOCX) [file pone.0238157.s008.docx]

**S4 Table**. **Details of candidate virulence genes in *Neonectria ditissima*.**

| Gene | SignalP^1^ - Predicted signal peptide | EffectorP^2^ | Predicted product size (amino acids)^3^ | |
| --- | --- | --- | --- | --- |
|  |  |  | +SP^4^ | - SP |
| *g4542* | Yes | 0.791 | 153 | 133 |
| *g5809* | Yes | 0.826 | 179 | 161 |
| *g7123* | Yes | 0.656 | 143 | 124 |

^1^ Software that indicates presence of secretion signal peptide [www.cbs.dtu.dk/services/SignalP/SignalP4.1](http://www.cbs.dtu.dk/services/SignalP/SignalP4.1)

^2^ EffectorP 2.0 software that indicates probability of being an effector protein.

^3^ Predicted amino acid sequence with and without signal peptide[www.cbs.dtu.dk/services/SignalP/SignalP4.1](http://www.cbs.dtu.dk/services/SignalP/SignalP4.1)

^4^ SP: signal peptide
