# Supplementary material for: Reference genes for gene expression analysis in the fungal pathogen Neonectria ditissima and their use demonstrating expression up-regulation of candidate virulence genes
Source: PLoS One. 2020 Nov 13;15(11):e0238157. doi: 10.1371/journal.pone.0238157 (PMC7665675; doi:10.1371/journal.pone.0238157)
Supplement: S5 Table — (DOCX) [file pone.0238157.s009.docx]

**S5 Table**. **Primer efficiency when amplifying candidate reference genes.**

| Gene | Average Cq | Slope | Efficiency (%) | SD | Amplification factor (E) | SD | R^2^ value* |
| --- | --- | --- | --- | --- | --- | --- | --- |
| *actin* | 18.64 | -3.409 | 96.74 | ± 1.871 | 1.967 | ± 0.01871 | 0.9940 |
| *Btub* | 22.50 | -3.157 | 107.53 | ± 3.985 | 2.075 | ± 0.03985 | 0.9959 |
| *mips* | 23.64 | -3.425 | 95.88 | ± 1.197 | 1.959 | ± 0.01197 | 0.9895 |
| *EfTu* | 21.63 | -3.785 | 83.74 | ± 0.653 | 1.8374 | ± 0.00653 | 0.9813 |
| *S8* | 21.53 | -3.553 | 91.17 | ± 2.246 | 1.9122 | ± 0.02246 | 0.9879 |
| *18sAMT* | 19.48 | -3.3116 | 100.43 | ± 3.499 | 2.0055 | ± 0.03499 | 0.9883 |

* Coefficient of correlation
