# Supplementary material for: Reference genes for gene expression analysis in the fungal pathogen Neonectria ditissima and their use demonstrating expression up-regulation of candidate virulence genes
Source: PLoS One. 2020 Nov 13;15(11):e0238157. doi: 10.1371/journal.pone.0238157 (PMC7665675; doi:10.1371/journal.pone.0238157)
Supplement: Attachment — Submitted filename: Response to Reviewers.docx [file pone.0238157.s010.docx]

Plant & Food Research
Private Bag 92169,

Auckland Mail Centre,

Auckland 1142,

New Zealand

15^th^ October, 2020

Dear Dr. Balestrini

**Re: PONE-D-20-24931: Reference genes for gene expression analysis in the fungal pathogen *Neonectria ditissima* and their use demonstrating expression up-regulation of candidate virulence genes**

We would like to thank you for the timely review of our manuscript and the suggestions from the reviewers for improvements.

In response to the Journal requirements we can confirm that:

- our manuscript meets PLOS ONE’s style requirements, including those for file naming.
- we are providing an original uncropped and unadjusted gel result file relating to the composite figure Fig 1 in the results named ‘S1_Fig’ which can be found in Supporting Information. The other supporting files have been renumbered.

We have addressed the comments from the Reviewers:

**In M&M please specify how the biological replicates were obtained.**

We have included (lines 128-132) the statement: One-year-old dormant *Malus* x *domestica* cv. ‘Royal Gala’ trees on rootstock ‘M793’ were inoculated with *N. ditissima*, isolate ICMP 23606, in September 2017. Forty-eight potted 1-year-old trees were arranged in a glasshouse, in a randomised block design, with four replicates, each comprising 12 trees. Of these 12 trees, six were inoculated (five inoculation sites per tree) and six mock-inoculated (three sites per tree), with sampling at six time points.

And at lines 145-147: Although four biological replicates (consisting of individual trees) were inoculated with pathogen or water for sampling at each time point, single inoculation sites were randomly selected from only three of the biological replicates for RNA isolation.

**In the results, I suggest separating the sections relating to the reference genes from the virulence genes. The paragraph starting on line 281 can be confusing for the reader. The authors can begin to describe the reference genes and the data for their selection, and at the end of the results they can introduce the virulence genes and their quantification.**

In the Results section, we have separated the results pertaining to reference gene selection from analysis of candidate virulence gene expression, so it is clearer for the reader. The virulence gene analysis starts once the results from the selection of reference genes is completed. For example the section originally from lines 289-302 has been moved to lines 381-395. The Results section is now split in to two; the first section is on the candidate reference genes with new subheadings within this section, and the second section is on candidate virulence genes, again with new subheadings.

**It would be interesting to compare in Fig 5 how the expression levels of the virulence genes change if they are normalized with the worst combination of reference genes. It would be interesting to show that the values are very different.**

We proceeded to do the comparison of the gene expression analysis of the virulence genes utilising the worst reference genes (*S8* and *Btub*) at two time points (week 6 and week 8 versus *in vitro*) for comparison with the data analysed using the two recommended reference genes. Using *S8* and *Btub* for normalisation results in a similar expression pattern of the candidate virulence genes to when *actin* and *mips* are used. This result is not unexpected since, for example, the geNorm M-value threshold analysis indicates that *S8* and *Btub* pass the M-value threshold for gene stability and so are still suitable for normalisation. However, the relative expression values are lower when the least stable reference gene combination is used and affects the statistical analysis of the significance of the differences between expression at different time points. This comparison has been added to the Methods, Results and Discussion sections as follows:

Methods line 273-274: The cDNA samples from 6 and 8 weeks post-inoculation were also analysed using the two least stably-expressed reference genes identified in this study.

Results line 445-452: Overall, the patterns of expression of the three candidate virulence genes were very similar when either the combination of the most stable reference genes *actin* and *mips*, or the two least stably expressed genes, *S8* and *Btub*, were used for normalisation. However, the relative expression values were greater when *actin* and *mips* were used for normalisation. Thus, when *S8* and Btub were used, differences in expression of the candidate virulence genes at different time points were deemed to be insignificant (p = 0.1231 to 0.6586, S4 Fig), where with *actin* and *mips* differences were significant (p < 0.001, S4 Fig). Moreover, expression *in planta* versus *in vitro* was not significantly different in *g5809* (p = 0.5477) and *g7123* (p = 0.5980) when using *S8* and *Btub*, whereas a significant difference can be observed when using *actin* and *mips* (p < 0.001, S4 Fig).

We have included a further figure in Supporting Information:

S4 Fig.	The relative expression of three *Neonectria ditissima* candidate virulence genes using (A) the least stable reference genes *S8* and *Btub* versus (B) the most stably expressed reference genes *actin* and *mips*. Data derived from three technical replicates from three biological replicates.

Discussion line 524-535: When expression levels of the candidate virulence genes at two time points were analysed using either the two least-stably expressed genes, *S8* and *Btub*, or *actin* and *mips*, the patterns of gene expression were similar, although relative expression levels were lower when *S8* and *Btub* were used. This is unsurprising since although they were the least-stably expressed, *S8* and *Btub* were classified as suitable to be used as reference genes by all the software packages. This phenomenon has also been observed in other studies with a decrease in expression when lower stability genes are used for normalisation (58, 59). However, more dramatic differences in expression levels can also be observed with significant alterations of relativities depending on the reference genes used (60). Although patterns of expression were retained, statistical analysis of the differences between the relative expression levels was affected, with a decrease in the significance of the differences between expressions at different time points *in planta* compared with expression *in vitro*. This indicates that use of the sub-optimal genes for normalisation may lead to nuanced differences in gene expression being overlooked.

**Throughout the manuscript please change qRT-PCR to RT-qPCR.** We have changed the abbreviation throughout the manuscript. We have also altered the definition of the abbreviation from ‘real-time quantitative reverse transcription PCR’ to ‘reverse transcription quantitative real-time PCR’, to re-inforce that the RT signifies reverse transcription rather than real-time.

In addition, following comments received after this manuscript was viewed on bioRxiv we have made a couple of further improvements:

Lines 139-140 and 143-144: We have changed the units for measuring temperatures during the pathogenicity tests from growing degree days to thermal time (°C days) as a more appropriate unit for standardisation of growing conditions between experiments.

Line 443: We have added the comment: There was no amplification of fungal sequences during RT-qPCR analysis when mock-inoculated cDNA was used as template throughout the time course.

We hope that the revisions we have made have addressed the points raised during the review process sufficiently to meet the requirements for publication in PLOS ONE.

Yours sincerely

Joanna Bowen
